# **The Effect of Steri-Strip Dressing on Patients’ Satisfaction and Reduction of Ecchymosis in Lower Eyelid, Malar and Cheek Following Rhinoplasty**

**Published:** 2016-01

**Authors:** Mohammad Reza Farahvash, Ghasemali Khorasani, Yadollah Mahdiani, Ahmad Reza Taheri

**Affiliations:** Department of Plastic Surgery, Tehran University of Medical Sciences, Tehran, Iran

**Keywords:** Rhinoplasty, Ecchymosis, Steri-strip, Subconjunctival bleeding, Satisfaction

## Abstract

**BACKGROUND:**

Early postoperative edema and ecchymosis are the most common factors to complicate initial patient perceptions about rhinoplasty**. **The current study was conducted to determine the effects of longer steri-strip tape on patient malar and cheek in terms of ecchymosis control and reduction.

**METHODS:**

Through a randomized controlled clinical trial, 64 patients who underwent rhinoplasty were randomly enrolled. One side of the patients’ face was randomly selected for different experience of dressing while the main intervention was different length of tape and steri-strip dressing. In one group, the right side and in the rest, the left side of face was applied with steri-stip from the nose to lateral malar and cheek. In the opposite side of the face, steri-strip taping was done from the nose to medical malar and cheek.

**RESULTS:**

The mean area of ecchymosis after rhinoplasty through our trial was 1.55 mm and 2.31 mm, respectively in sides with and without steri-strip which differed significantly. When patients’ age and sex were taken into account, the distribution of ecchymosis had no significant difference in this regard.

**CONCLUSION:**

The present study showed significant reduction in the area of post-rhinoplasty ecchymosis in lower lid, malar and cheek soft tissues as well as the obvious increase in satisfaction rate among intervention side of face in comparison to the control side. But longer steri-strip tape failed to control sub conjunctival bleeding or decrease it.

## INTRODUCTION

Early postoperative edema and ecchymosis are the most common factors to complicate initial patient perceptions about rhinoplasty.^1^^-^^4^ Many people express different emotions like anxiety, worry and even anger when observing the postoperative changes which are usually normal to occur. “Post-rhinoplasty dissatisfaction syndrome” may be the most predominant problem in this regard.^5^ Authorities have explained the fact that apart from physical consequences of post-rhinoplasty dressing, tape placing over the nose provides a condition to help patient adjust to the new appearance, psychologically when have a partial view of his or her new nose.^1^

For the patient, swelling and ecchymosis play the most important role in satisfaction rate and self-confidence after rhinoplasty which are, in turn, resulted by osteotomies through the procedure because of the injured angular vessels which cross the site of osteotomy and fractured bone, in details.^6^^-^^12^ The special situation of post rhinoplasty edema and ecchymosis has made the named complications a timeless factor to focus on to find several ways to reduce them. Many researchers and authorities have attempted in this regard to advise following approaches: (i) To use local vasoconstrictive solutions at the site of surgery, (ii) To use micro-osteotomes as rather newly developed tools, (iii) To preserve periosteal attachment, (iv) To prevent transverse osteotomy by medial-oblique approach, and (v) Steroid administration.^11^^-^^13^

The latter item addresses to the effect of glucocorticoids on decreasing vascular permeability to lessen the local exudation and blood accumulation at the site of manipulation.^8^ In spite of the vast number of performances to introduce new ways to control postoperative edema and ecchymosis, there has been no research to work on compressive effects of steri-strip tape on the bleeding sites following rhinoplasty, as far as we know. So, the current study headed to find the results of longer steri-strip tape on patient cheek in terms of edema and ecchymosis control and reduction.

## MATERIALS AND METHODS

Through a randomized controlled clinical trial, 64 patients who had undergone rhinoplasty were randomly selected to enter the study. Smokers as well as people with hemostatic problems, hypertension and previous rhinoplasty experience were excluded from the study. One side of the patients’ faces were randomly selected to get different length of dressing named Steri-Strip. In one group, the right side and in the rest, the left side of face had steri-stip dressing which was a longer tape application from the nose to lateral malar and cheek. 

The opposite side had shortey conventional tape dressing from 3 the nose to medial malar and cheek. The participants were followed up for 2 months to assess the outcome of rhinoplasty as well as the complications among which ecchymosis in addition to sub- conjunctival bleeding were most predominant. One week after the surgery, at the first postoperative physician visit, 3M tapes and nasal splints were removed before using a digital camera to get facial photographs at face and bilateral views. Strict protocol was applied to have real size photographs to be compared. A 6 megapixels digital camera was placed in 2 meters far from the subject at 150 centimeters above the floor. The subjects sat on a chair and were fixed at the vertical level of the camera. 

**Fig. 1:**
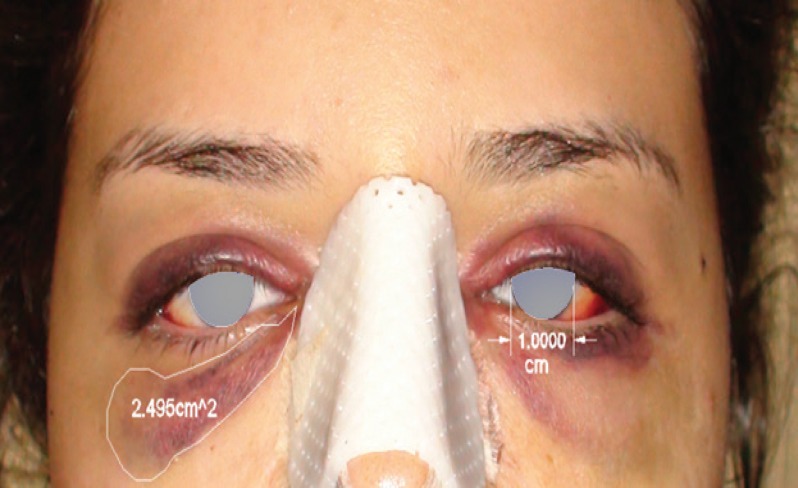
Two computer soft wares namely, “Autocad” and “Image 2cad” were used to compute ecchymosis area in lower lid, malar and cheek parts of face bilaterally in cm^2^

The light and contrasts were considered to be the same for all photographs and the pictures were captured by the same skilled medical photographer. Two computer soft wares namely, “Autocad” and “Image 2cad” were used to compute ecchymosis area in lower lid, malar and cheek parts of face bilaterally in cm^2 ^(Figure 1). To prevent any size or distant noncompliance and errors, Autocad special tools helped us adjust the diameter of the iris 1 cm for all patients. This was a great step to control the least errors before any comparison based on the fact that the diameter of the iris is 1 cm in average in the majority of human societies.

This is strongly worth pointing out that all the surgeries were done by the same surgical team and the areas of complications were assessed by a blind computer operator for both control and intervention sides of patients’ faces. Generally, the study was conducted as a double blind research and the sample size was estimated 10% more than the exact computed number to prevent the effects of the loss patients during follow up. The other focused field in this study was patient satisfaction. This satisfaction was just regarding his/her appearance especially about local ecchymosis and bleeding. 

The patients gave their opinions by an alphabetical order. Letter “A” was for cases who accepted their appearance, meaning high satisfaction. The patients who had expected less complication occurrence gave “B” to their experience while people who worried about the outcome considered “C”. All the patients used cold compress at the operation site within the first 48hrs after the surgery. External osteotomy by sharp 2-3 mm osteotomes was done while the skin was not stitched up at the entry site of the osteotomes. All the participants took 8 mg intravenous dexamethasone and 100 mg of hydrocortisone immediately before rhinoplasty to prevent severe inflammation and bruise.

The confidence interval was considered 95% with a type one error α=0.05 and the significance=0.05. SPSS 18 for windows was utilized through Chi Square and t tests. This study reported only the frequencies of the studied items via a split-study in face. The participants were not avoided from any conventional and classic procedure; but they just experience longer tape dressing on one side of their face. No facilities were omitted for the people who disagreed to take part in this study.

## RESULTS

Totally, 64 subjects were enrolled in the study including 11 (17.2%) males and 53 (82.8%) females as can be seen in Table 1. The mean area of ecchymosis after rhinoplasty, through our trial, was 1.55 cm^2^ and 2.31 cm^2^, respectively in sides with and without steri-strip which differed significantly (*p*=0.001). When patients’ sex was taken into account, Table 2 shows the distribution of ecchymosis considering the new factor with no significant difference in this regard. 

**Table 1:** Sex distribution between the studied patients

**Sex distribution**	**Number**	**Percent**
Male	11	17.2
Female	53	82.8
Total	64	100.0

**Table 2:** Ecchymosis area considering patients’ gender

	**Significance**	**Without Steri-Strip (cm2)**	**With Steri-Strip (cm2)**
		**0.07**	**0.1**
Male	Mean	1.9163	1.4906
	SD	1.25050	1.22334
Female	Mean	2.8378	2.0124
	SD	1.58690	1.03849
Total	Mean	2.6794	1.9227
	SD	1.56510	1.08031

The participants were divided into two groups regarding their age containing <30 years and >30 years old. Likely, age was not a factor of difference between the patients concerning the mean area of postoperative ecchymosis as Table 3 summarizes. Sub-conjunctival bleeding was the other important evaluated complication of rhinoplasty throughout this study. Finally, 25% and 21.9% of bleeding were reported in sides without and with steri-strip, respectively among which no statistical significant difference was found (Table 4).

**Table 3:** Ecchymosis area regarding participant’s age group

**Age group**		**Without Steri-Strip (cm2)**	**With Steri-Strip (cm2)**
	**Significance**	**0.7**	**0.7**
<30	Mean	2.7580	1.9833
	SD	1.49416	1.17710
>30	Mean	2.6220	1.8785
	SD	1.63289	1.01830
Total	Mean	2.6794	1.9227
	SD	1.56510	1.08031

**Table 4:** The occurrence rate of subconjunctival bleeding

	**Without Steri-strip**		**With Steri-strip**	
	**Count**	**%**	**Count**	**%**
Bleeding	16	25.0	14	21.9
No Bleeding	48	75.0	50	78.1
Total	64	100.0	64	100.0

We assessed the participants’ satisfaction by dividing them into three levels of low, intermediate and high satisfaction. Forty eight (75%) patients expressed their high satisfaction while 11 (17.2%) and 5 (7.8%) had intermediate and low rates of satisfaction, especially in terms of predominant complications like ecchymosis and sub conjunctival bleeding. (Table 5).

**Table 5:** Satisfaction rates in participants

	**Count**	**%**
High satisfaction	48	75.0
Intermediate satisfaction	11	17.2
Low satisfaction	5	7.8
Total	64	100.0

**Fig. 2:**
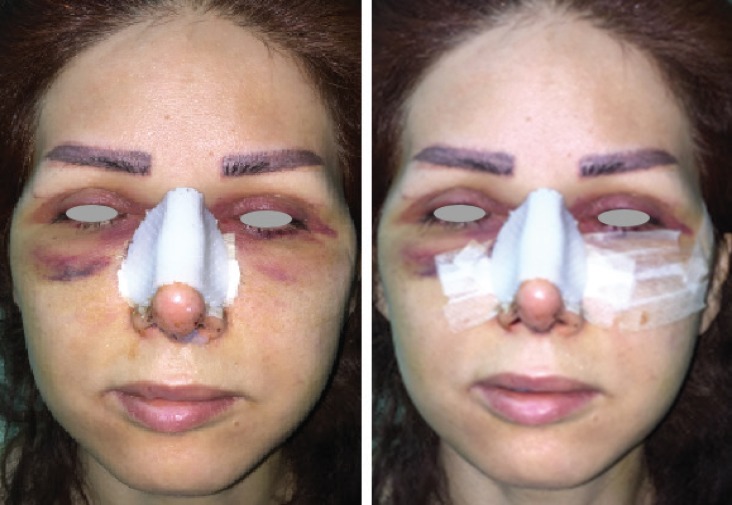
Success of steri-strip tape for controlling post rhinoplasty ecchymosis

The present study showed significant reduction in the area of post-rhinoplasty ecchymosis in lower lid, malar and cheek soft tissues as well as the obvious increase in satisfaction rate among intervention side of face in comparison to the control side. We found that longer steri-strip tape failed to control sub conjunctival bleeding or decrease it (Figure 2).

## DISCUSSION

The present study tried to compare the conventional short tape dressing and long steri-strip tape dressing following rhinoplasty in the prevention or decrease of postoperative ecchymosis which usually occur and disturbs the rate of satisfaction in patients and surgeons. The results showed a significant difference of ecchymosis area between two sides of patients’ faces. Longer tapes had obviously less ecchymosis that confirmed the helpful role of compression on cheek and nasal bone in this matter. Edema and ecchymosis, despite of the usual gentle and careful performances by even the most expert surgeons, are inevitable and could strongly affect the patient and surgeon satisfaction, physically and mentally. ^14^

The edema and ecchymosis, which develop around the eyes after rhinoplasty, have been focused by many studies for decades and some techniques have been found to improve compression and dressing techniques in this matter. Many medications are usually used in cosmetic operations, particularly in face such as adrenaline, arnica, lidocaine, and corticosteroids as well as some herbal extracts. ^15^^-^^19^ Some also used hypotensive drugs during head and neck surgeries ^20^^,^^21^ which are not systematically offensive to be used only to reduce ecchymosis.

In a clinical trial by Simsek et al. in Turkey in 2011, two surgical techniques of rhinoplasty with and without osteotomy showed significantly different size and rate of ecchymosis in the patients which suggest the superiority of rhinoplasty without osteotomy in terms of ecchymosis occurrence. ^22^ This confirms that osteotomy; especially transverse osteotomy could potentially direct patients to get edema and ecchymosis following the procedure. However, since all the patients enrolled in our study experienced osteotomy, we through the current trial, tried to assess the effects of simply different dressing techniques on the size of ecchymosis while used in addition to perioperative corticosteroids as the conventional way of control the common complications.

As pointed out before, we failed to find any attempts in the literature to assess the compressive aspects of tape and cast to control ecchymosis. As it turns out, there is some herbal extracts which are systematically administered to reduce the edema and ecchymosis following rhinoplasty successfully as Chaiet et al. tried well. ^23^ Concerning steroids and their reducer effects on edema and ecchymosis, these medications, with several drug shapes, have been the most group to be studied and used conventionally after surgeries, especially in face, head and neck. Hwang et al. were one of the groups who published even meta-analysis in this matter in 2014.^24^ They determined that through several studies found in the literature, in more than total 300 patients with rhinoplasty, upper eyelid ecchymosis was significantly decreased by using steroids when compared with the control group. Lower eyelid ecchymosis decreased significantly in the assessed studies but the effect size of steroids in upper eyelid was reported higher. They also advised using multiple doses of steroids instead of single dose to achieve a better control in edema and ecchymosis. 

We found no effect of age and sex on the rate and size of ecchymosis after rhinoplasty during our trial. Likely, no study explained sex or age different distribution of the named complication. We also found no significant effectiveness of long tape on sub conjunctival bleeding when comparing the intervention and control sides of patients’ faces. This is while Kosucu et al. in Turkey showed significant reduction of bleeding by using hypotensive drug, remifentanil during rhinoplasty. ^14^

The present split-clinical trial on patients’ faces provides a reliable way to compare the net effect of different length of steri-strip tapes because of the same physical and physiological base of individuals who each of them was separately a case and at the same time a control. There would be an advice to use long steri-strip tapes from the nose to lateral malar and cheek in both side of the fase in rhinoplasty patients along with topical platelet gels when dressing the operated nose to allow this autologous and homogenous platelet-rich and fibrin-rich gel attract inflammatory cells as well as fibroblasts to stimulate collagen local deposition to prevent or decrease the size of bleeding and ecchymosis following surgery. ^25^^-^^28^

To conclude, the present study showed significant reduction in the area of post-rhinoplasty ecchymosis in lower lid, malar and cheek soft tissues as well as the obvious increase in satisfaction rate among intervention side of face in comparison to the control side. But longer steri-strip tape failed to control sub conjunctival bleeding or decrease it.

## CONFLICT OF INTEREST

The authors declare no conflict of interest.
